# Regulation of Angiogenesis by Aminoacyl-tRNA Synthetases

**DOI:** 10.3390/ijms151223725

**Published:** 2014-12-19

**Authors:** Adam C. Mirando, Christopher S. Francklyn, Karen M. Lounsbury

**Affiliations:** 1 Department of Biochemistry, College of Medicine, University of Vermont, Burlington, VT 05405, USA; E-Mails: adam.mirando@uvm.edu (A.C.M.); Christopher.Francklyn@uvm.edu (C.S.F.); 2 Department of Pharmacology, College of Medicine, University of Vermont, Burlington, VT 05405, USA

**Keywords:** angiogenesis, aminoacyl-tRNA synthetase, endothelial cells, non-canonical functions

## Abstract

In addition to their canonical roles in translation the aminoacyl-tRNA synthetases (ARSs) have developed secondary functions over the course of evolution. Many of these activities are associated with cellular survival and nutritional stress responses essential for homeostatic processes in higher eukaryotes. In particular, six ARSs and one associated factor have documented functions in angiogenesis. However, despite their connection to this process, the ARSs are mechanistically distinct and exhibit a range of positive or negative effects on aspects of endothelial cell migration, proliferation, and survival. This variability is achieved through the appearance of appended domains and interplay with inflammatory pathways not found in prokaryotic systems. Complete knowledge of the non-canonical functions of ARSs is necessary to understand the mechanisms underlying the physiological regulation of angiogenesis.

## 1. Introduction

The aminoacyl-tRNA synthetases (ARSs) are an ancient and ubiquitous family of enzymes essential for the synthesis and screening of charged-tRNA for use in translation. The reaction proceeds through a two-step mechanism in which an aminoacyl-adenylate is formed through condensation of the amino acid with ATP followed by transfer of the amino acid to the terminal adenosine moiety of the corresponding tRNA. However, in addition to their canonical functions, a growing body of evidence has identified several secondary activities associated with ARSs. Among the earlier reported non-translational functions were effects on RNA splicing. Mitochondrial varieties of tyrosyl-tRNA synthetase and leucyl-tRNA synthetase possess splice factor-like activities and assist in the excision of group I introns [1,2]. Transcriptional and translational regulatory properties have also been identified in ARSs through association with transcription factors or the binding of tRNA-like structures within mRNA promoter regions [3,4]. Furthermore, ARSs in higher-order eukaryotes have evolved roles in both internal and external cellular signaling responses, including apoptosis and inflammation [5,6]. However, this review will focus on the observation that several ARSs demonstrate the ability to influence various aspects of angiogenesis.

Angiogenesis, the growth of new blood vessels from preexisting ones, is a multifaceted process governed by the balance of pro-angiogenic and quiescent signals. The factors mediating these effects consist of various extracellular matrix (ECM) components, soluble growth factors, cell–cell adhesion proteins, and associated receptors [7,8]. Under normal conditions vessels maintain a quiescent state through the release of angiopoietin-1 and other survival signals from mural cells [9]. However, the spatially and temporally regulated expression of other factors can tip the balance positively or negatively with respect to proliferation, migration, and maturation of nascent vasculature. Many of these factors, such as fibroblast growth factor (FGF) and platelet-derived growth factor (PDGF), are functionally redundant and associated with a variety of biological functions [9]. Conversely, vascular endothelial growth factor A (VEGFA, originally vascular permeability factor) and its corresponding receptor, VEGFR2 (also referred to as the kinase insert domain receptor (KDR) or fetal liver kinase 1 (Flk1)), are indispensable for the process of angiogenesis and are highly specific to endothelial cell signaling [10]. VEGFA is released from tissues in response to hypoxia, inflammation, or wound healing and activates VEGFR2 receptor tyrosine kinase activity in endothelial cells. Consequently, proliferation, migration, and survival are enhanced by the activation of downstream signaling targets, including ERK1/2, p38-MAPK, and AKT [11]. While these various kinases may elicit overlapping effects, their differences allow for the fine tuning of the angiogenesis process by favoring specific responses, such as migration or proliferation.

To date, angiogenesis-associated activities have been observed in tyrosyl-tRNA synthetase (YARS), tryptophanyl-tRNA synthetase (WARS), threonyl-tRNA synthetase (TARS), seryl-tRNA synthetase (SARS), glutamyl-prolyl-tRNA synthetase (EPRS) and one closely associated protein, aminoacyl-tRNA synthetase interacting multifunctional protein 1 (AIMP1, also known as p43) (Table 1). Similar to growth factors, YARS, WARS, TARS, and AIMP1 function extracellularly, and thought to be secreted. Once outside the cell, receptor interactions vary with each ARS and elicit very different responses related to inflammation, proliferation, migration, and cell-cell contacts. Alternatively, SARS and EPRS regulate angiogenesis intracellularly through interactions with DNA and mRNA associated with the transcription and translation of VEGF and other angiogenic genes. While this variety of mechanisms complicates our understanding of ARS secondary function, it also opens up opportunities for the development of drugs targeting specific aspects of angiogenesis. Therefore, further progress in this field would improve our knowledge of mammalian physiology and provide new therapeutic targets for vascular-based therapies.

**Table 1:** Angiogenesis-associated activities of aminoacyl-tRNA synthetases. Aminoacyl-tRNA synthetases (ARSs) are subdivided based on whether they elicit angiogenesis-based responses outside the cell, inside the cell, or are ARS-associated proteins. Effects on angiogenesis are reported as “Pro” for pro-angiogenic responses (*i.e*., stimulate new blood vessel growth), “static” for angiostatic responses (*i.e.*, inhibit new blood vessel growth), and “Anti” for anti-angiogenic responses (*i.e.*, reduce current number of vessels).

ARS or Associated Factor	Location of Angiogenic Function	Effect on Angiogenesis	Associated Domains	Details
**Externally Acting ARS**				
YARS	Extracellular	Pro	ELR motif, EMAP II-like [12,13]	Secreted and cleaved into N and C cytokine fragments; *N*-terminal ELR stimulates angiogenesis; *C*-terminus may possess EMAP II-like signaling [12,13,14,15]
WARS	Extracellular	Static	WHEP domain [16]	Secreted and WHEP-domain removed by secretion or alternative splicing; loss of WHEP-domain allows for interactions with E-cadherin [16,17,18,19]
TARS	Extracellular	Pro	Catalytic domain (possibly others) [20]	Secreted and stimulates vessel migration, patterning, and maturation; Based on borrelidin binding site, the mechanism is likely dependent, at least partially, on the catalytic domain [20,21]
**Internally Acting ARS**				
SARS	Nucleus	Static	NLS [22,23,24]	Directed to nucleus by NLS; Binds to *vegfaa* promoter and disrupts cMyc induction of *vegfaa* mRNA [22,23,24]
EPRS	Cytoplasm	Static	Three WHEP domains [25]	IFNγ stimulates the release of EPRS from the MSC and its association with the GAIT complex; complex binds to mRNA 3' elements and inhibits translation [25,26,27,28,29,30,31,32]
**ARS Associated Factors**				
AIMP1	Extracellular	Pro (low conc.) Anti (high conc.)	EMAP II [33]	Released from MSC in response to apoptotic signals; secreted as full-length AIMP1 or EMAP II; stimulates angiogensis at low concentrations but induces EC apoptosis at high concentrations [33,34,35,36,37,38,39,40,41]

AIMP1: Aminoacyl-tRNA synthetase interacting multifunctional protein 1; ARS: Aminoacyl-tRNA synthetases; EC: Endothelial cell; ELR: Glutamate-leucine-arginine sequence motif; EMAP II: endothelial monocyte activating polypeptide II; EPRS: Glutamyl-prolyl-tRNA synthetase; GAIT: interfere-γ-activated inhibitor of translation; IFNγ: Interferon-γ; NLS: Nuclear localization signal; SARS: Seryl-tRNA synthetase; TARS: Threonyl-tRNA synthetase; WARS: Tryptophanyl-tRNA synthetase; WHEP-domain: A unique domain named or its association with tryptophanyl-, histidyl-, and glutamyl-prolyl-tRNA synthetases; YARS: Tyrosyl-tRNA synthetase.

## 2. Regulation of Angiogenesis by Aminoacyl-tRNA Synthetases

### 2.1. Angiogenic Signaling by Extracellular ARSs

**Figure 1:**
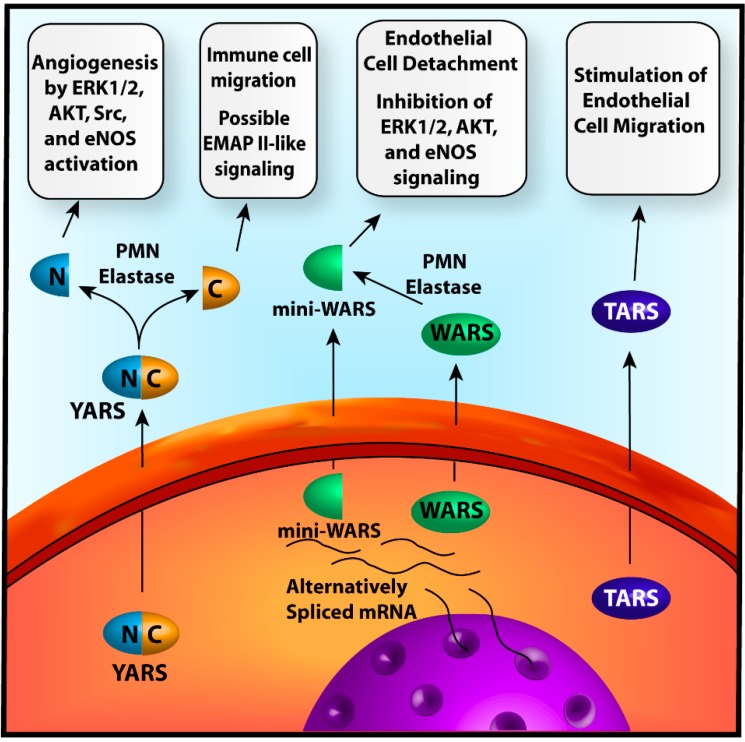
Mechanisms of angiogenesis by extracellularly acting ARS. *Left*-Full-length tyrosyl-tRNA synthetase (YARS) is secreted from the cell by an unknown mechanism. Outside the cell it is cleaved by polymorphonuclear leukocyte (PMN)-elastase or other protease molecules into *N*- and *C*-terminal fragments which stimulate angiogenesis or immune responses respectively; *Center*-Full-length tryptophanyl-tRNA synthetase (WARS) or mini-WARS are generated from alternative splicing of WARS mRNA and are subsequently secreted into the extracellular space by unknown mechanisms. Full-length WARS is further processed by PMN-elastase or other proteases to form fragments like mini-WARS. The WARS fragments disrupt endothelial cell–cell contacts and angiogenic signaling molecules, eliciting an angiostatic effect; *Right*-Full-length threonyl-tRNA synthetase (TARS) is secreted from the cell by unknown processes and stimulates vessel migration. The mechanisms of these effects have yet to be determined.

The process of angiogenesis requires the collaborative effort of numerous cells and cell-types. Coordination of their activities relies heavily on the secretion of various chemokines and growth factors to disseminate information throughout the local microenvironment. The balance of these factors determines the overall stimulatory or inhibitory nature of the signals [9]. Three of the angiogenesis-associated ARSs, YARS, WARS, and TARS, utilize a similar mechanism in which the enzymes are secreted from the cell in response to certain stimuli to engage in autocrine and paracrine type signaling (Figure 1).

#### 2.1.1. Tyrosyl-tRNA Synthetase

The angiogenic properties of YARS are mediated by pro-inflammatory features that were incorporated into the protein during the evolution of higher eukaryotes. Early sequencing of YARS revealed an appended *C*-terminal domain in eukaryotes that shares 40% homology with endothelial monocyte activating polypeptide II (EMAP II), a pro-cytokine that is released from cells in response to apoptotic signals [12]. Under similar conditions, YARS is secreted into the extracellular space. While no signaling activity is observed for the full length protein, cleavage by extracellular polymorphonuclear leukocyte (PMN) elastase generates two separate fragments: an *N*-terminal catalytic domain fragment, termed “mini-YARS”, and a *C*-terminal EMAP II-like domain. Similar to EMAP II, the *C*-terminal domain promotes chemotaxis in immune cells and stimulates the release of tumor necrosis factor-α (TNFα) and tissue factor cytokines. While no direct connection between angiogenesis and the YARS *C*-terminal domain has been reported, EMAP II itself originates from an ARS associated protein related to angiogenesis and will be discussed in greater detail below. Interestingly, exposure to the mini-YARS fragment also elicits chemotactic responses in leukocytes similar to α-chemokines (Cys-X-Cys (CXC)-chemokines), suggesting that it possesses cytokine activity as well [13]. Therefore, the rest of this section will focus on the mini-YARS fragment.

Like other members of the CXC-chemokine family, mini-YARS possesses a highly conserved Glu-Leu-Arg (ELR) motif essential for receptor interactions. Scatchard analysis of ^125^I-labeled mini-YARS binding to PMNs revealed saturable binding, indicative of a specific receptor. This interaction is not observed in mutants with a Glu-Leu-Gln (ELQ) sequence suggesting that the association is dependent on an intact ELR motif. Furthermore, this binding is susceptible to competition by increasing amounts of the CXC-chemokine interleukin-8 (IL-8), suggesting that the IL-8 receptor type A (CXCR1) is the likely mini-YARS receptor. These results are supported by the observation that mini-YARS bound to cells transfected with the gene for CXCR1 but not those with the CXCR2 gene [13]. Although these studies focused on YARS signaling in immune cells, the ELR-motif bearing CXC chemokines also exhibit pro-angiogenic activities [14].

The angiogenic potential of mini-YARS has been investigated through a battery of *in vitro* and *in vivo* studies. Similar to other CXC-chemokines, mini-YARS induces pro-angiogenic responses related to endothelial cell migration and proliferation. Transwell migration assays indicate a significant increase in the migration of endothelial cells when mini-YARS is present in the lower chamber [14,42]. Similarly, endothelial cell cultures treated with mini-YARS show increased migration of cells to scratch sites in wound migration assays. Together, these studies suggest that YARS invokes chemotactic responses in endothelial cells similar to those observed with immune cells. Additionally, treatment of endothelial cells with mini-YARS stimulates proliferation and organization of vessel networks according to commercial dye and tube formation assays respectively. Interestingly, the extent of these angiogenic effects by mini-YARS is comparable to that demonstrated by VEGF, indicating a potent response [42]. These observations are supported by several *in vivo* models as well. Exposure to mini-YARS increases basal vessel formation in chorioallantoic membrane (CAM) and mouse matrigel models of angiogenesis [14].

The pro-angiogenic responses observed for the migration, tube-formation, and CAM assays are dependent on an intact ELR motif as mutation of any of these residues in mini-YARS appears to inhibit these processes. Given the importance of the ELR for the binding of mini-YARS to CXCR1, these results implicate this receptor as the mediator of angiogenesis. While there was originally some doubt of this due to the lack of a murine CXCR1 homolog, subsequent studies have since identified a potential rodent CXCR1 candidate [43], suggesting that this receptor pathway may still be viable in mouse models. In addition, similar angiogenic effects demonstrated by mini-YARS and VEGF led investigators to examine the involvement of another receptor, VEGFR2. Treatment with mini-YARS stimulates phosphorylation of Y1054, activating the receptor. Because VEGF expression is not stimulated by exposure to mini-YARS, the authors believe that signaling by the ARS activates VEGFR2 through a trans-activation mechanism.

Investigation of down-stream mini-YARS signaling cascades revealed phosphorylation of ERK, Src, and AKT which have been previously demonstrated to initiate pro-angiogenic responses endothelial cell migration, proliferation, and survival [15]. To further investigate the importance of ERK signaling, the downstream kinase MEK was inhibited using the compound U0126. Interestingly, this treatment blocks mini-YARS induced migration, suggesting that the ERK signaling cascade is important for mini-YARS mediated angiogenesis. In addition to kinases, mini-YARS activates endothelial nitric oxide synthase (eNOS) through phosphorylation of Ser-1179, leading to increased nitric oxide (NO) production. Previous studies have demonstrated significant effects by NO on vascular permeability and other angiogenic responses, suggesting that it could be another contributing factor to YARS’s pro-angiogenic activity [44]. Overall, the combination of these various signaling cascades establishes a clear angiogenic mechanism for extra-cellular YARS that is characteristic of its similarities to the CXC-chemokine family.

#### 2.1.2. Tryptophanyl-tRNA Synthetase

Aspects of WARS secondary functions were observed as early as 1969 with the appearance of alternatively spliced fragments in preparations from bovine pancreas extracts [45]. Later, the presence of WARS within exocrine cells provided evidence of secretion [46]. Subsequently, a truncated, alternative splice-form of WARS, termed mini-WARS, was discovered. Interestingly, the structure of the WARS catalytic core closely resembles that of YARS and the discovery of pro-angiogenic functions by fragments of YARS indicates that WARS fragments may also possess possible biological functions separate from aminoacylation [17]. Some of the general mechanisms governing WARS’s secondary functions appear similar to YARS; however, the two proteins elicit opposing responses on angiogenesis. Expression of both mini-WARS and the full-length protein is regulated by interferon-γ (IFNγ), a cytokine that stimulates the expression of angiostatic chemokines and suppresses the expression of the pro-angiogenic cytokine IL-8. This correlation between the structures of WARS and YARS coupled with the biological connection with IFNγ led to investigations of potential angiogenic effects of WARS.

Like YARS, full-length WARS has no influence on endothelial cell migration and proliferation assays prior to truncation of the appended WHEP domain. This can be accomplished through alternative splicing, as in mini-WARS, or through limited proteolysis, such as by PMN elastase, which generates T1 and T2 WARS fragments. However, whereas the active YARS fragments are pro-angiogenic, WARS fragments inhibit both VEGF- and YARS-induced migration and proliferation in endothelial cells, suggesting an angiostatic function [14,17]. Similar attenuation of angiogenic responses are observed in the CAM, mouse matrigel plug, and mouse retinal models indicating that these effects can be recapitulated under *in vivo* conditions.

To better understand the inhibitory effects of WARS, the effect of the T2-WARS fragment on downstream, angiogenic kinase cascades was investigated. The presence of extracellular, T2-WARS inhibits the VEGF- and shear stress-mediated phosphorylation of ERK, AKT, and eNOS [18,19]. The localization of T2-WARS to the cell periphery [18] coupled with the susceptibility of treated endothelial cells to shear induced detachment [19] led to the hypothesis that WARS fragments interacted with cell-cell contacts. Subsequently it was determined that knockdown of vascular endothelial cadherin (VE-cadherin) disrupts the interaction between WARS and endothelial cells, implicating it as the putative extracellular mediator of WARS’s non-canonical activities [18]. Molecular modeling studies suggest that the basis of this interaction was the insertion of Trp2 and Trp4 from the *N*-terminal EC1 domain of VE-cadherin into the active site of the WARS core. This is supported by mutational analyses in which substitution of Trp2 and Trp4 on EC1 or essential binding residues in WARS abolish the interaction between the proteins. Interestingly, the binding of VE-cadherin to full-length WARS is occluded by the *N*-terminally appended WHEP domain in WARS. These helix-turn-helix domains are exclusive to ARSs and are named for tryptophanyl (W), histidyl (H), and glutamlyl-prolyl (EP) ARSs in which they are found. The loss of this domain in WARS fragments allows VE-cadherin to access the WARS active site, providing a mechanism for the cleavage-dependence of WARS’s anti-angiogenic activity [16].

As with both YARS and TARS (see below), the mechanism of WARS exocytosis remains unknown. The fact that WARS-secretion is not affected by brefeldin A or A23187 indicates that it is not dependent on Golgi- or calcium-dependent transport respectively [47]. The lack of a canonical export sequence led to the hypothesis that secretion may be dependent on protein-protein interactions. Yeast two-hybrid and co-immunoprecipitation experiments revealed an interaction between WARS and p11 (also referred to as S100A10), a protein member of the annexin II complex [47]. Previously p11 had been found to be important for the cellular retention of the TASK-1 transcription factor [48]. Similarly, the connection of this complex to WARS’s retention was confirmed by demonstrating that knockdown of either p11 or annexin II increases the secretion of WARS [47]. Taken together, these data suggest a model in which angiostatic signals stimulate the release of WARS from p11 allowing for its subsequent secretion from the cell through unknown mechanisms. Once in the extra-cellular milieu WARS can then be processed into active fragments that inhibit angiogenesis by disrupting endothelial cell-cell contacts.

#### 2.1.3. Threonyl-tRNA Synthetase

Much of our understanding of TARS-mediated angiogenesis was obtained concurrently with studies of the TARS specific inhibitor borrelidin, a naturally occurring polyketide anti-microbial and anti-fungal compound from *Streptomyces rochei*. Characterizations of borrelidin-resistant bacterial strains and Chinese hamster ovary cells with selective amplification of *tars* genes established TARS as the principal target of borrelidin [49,50]. Subsequent investigations revealed that borrelidin inhibits tube formation of rat aortic ring fragments with an IC_50_ of 0.4 ng/mL (0.8 nM). This value was 15-fold lower than the IC_50_ for endothelial cell growth inhibition (6 ng/mL), suggesting a specific anti-angiogenic activity for the compound [51]. The fact that no alternative targets to TARS can be identified coupled with the threonine-dependent nature of some of the anti-angiogenic effects strongly implicates TARS as the mediator of these effects. However, it was not until recent investigations using a borrelidin-resistant point mutant that the TARS-dependent nature of this activity was confirmed [21].

Exposure of endothelial cell cultures and CAM assays to recombinant TARS produces significant increases in both tube formation and basal CAM vessel formation respectively, comparable to the results observed for the VEGF controls. In both types of assays, the angiogenic effects are susceptible to inhibition by BC194, a less toxic derivative of borrelidin. Interestingly, the concentration of BC194 used in the assays, 10 nM, is below the threshold concentrations demonstrated to induce phosphorylation of eIF2α and the cleavage of caspase 3 [21]. Phospho-eIF2α is a downstream marker of the general control nonderepressible 2 (GCN2)-mediated amino acid starvation associated with the accumulation of uncharged tRNA. Sufficient inhibition of the canonical aminoacylation in ARSs is known to stimulate this response [52]; therefore, the lack of eIF2α phosphorylation suggests that the inhibition of angiogenesis is not the result of loss of cell viability from inhibition of translation. This conclusion is also supported by experiments demonstrating that treatment with BC194 does not inhibit total protein translation in cells as compared to the ribosome inhibitor cycloheximide [21]. Likewise, cleaved-caspase 3 is an indicator of apoptotic signaling and has been previously associated with borrelidin-mediated endothelial cell death [53]. Its absence at 10 nM BC194 suggests that the above effects are specifically related to a TARS-angiogenic function not associated with cell death and toxicity pathways.

The TARS specificity was examined by creating L567V TARS, a BC194-resistant mutant that exhibits a nearly 8-fold higher *K*_i_ relative to wildtype TARS. While the mutant was able to induce angiogenesis comparably to wildtype in both endothelial cell tube formation and CAM assays, BC194 treatment has no effect on L567V-induced angiogenesis, confirming that BC194’s anti-angiogenic effect is TARS-dependent in its role in translation. These data are consistent with earlier investigations of *E. coli* TARS, in which mutations within the same region of the enzyme influenced its *K*_i_ for borrelidin [20]. Together, these data suggest that the catalytic domain is, at least in part, responsible for TARS’s non-canonical activity.

In the above assays, recombinant TARS stimulates angiogenesis when applied extracellularly. This finding suggests that TARS may be secreted and interact with surface receptors in response to specific stimuli, much like YARS and WARS. In support of this model, conditioned media from endothelial cells is found to contain small levels of TARS. Treatment of the cells with the pro-angiogenic cytokines TNFα and VEGF significantly increased the levels of TARS in the conditioned media, indicating that TARS secretion is induced during angiogenesis and may function in autocrine signaling. Additionally, treatment with TNFα has been demonstrated to stimulate secretion of TARS from SKOV-3 ovarian carcinoma cells, suggesting a network of paracrine signaling in a tumor microenvironment and, possibly, normal tissues as well [54].

Despite knowledge of its secretion the mechanism of extracellular TARS-mediated angiogenesis remains to be fully elucidated. Alamar blue assays demonstrated that recombinant TARS had no effect on the proliferation of endothelial cell cultures. Conversely, when placed in the lower chamber of a transwell migration assay, TARS stimulated endothelial cell migration [21]. Taken together, these data indicate a role for extracellular TARS in the migration of growing vasculature through yet unknown molecular mechanisms.

### 2.2. Transcriptional and Translational Regulation of Angiogenesis by Aminoacyl-tRNA Synthetases (ARSs)

**Figure 2:**
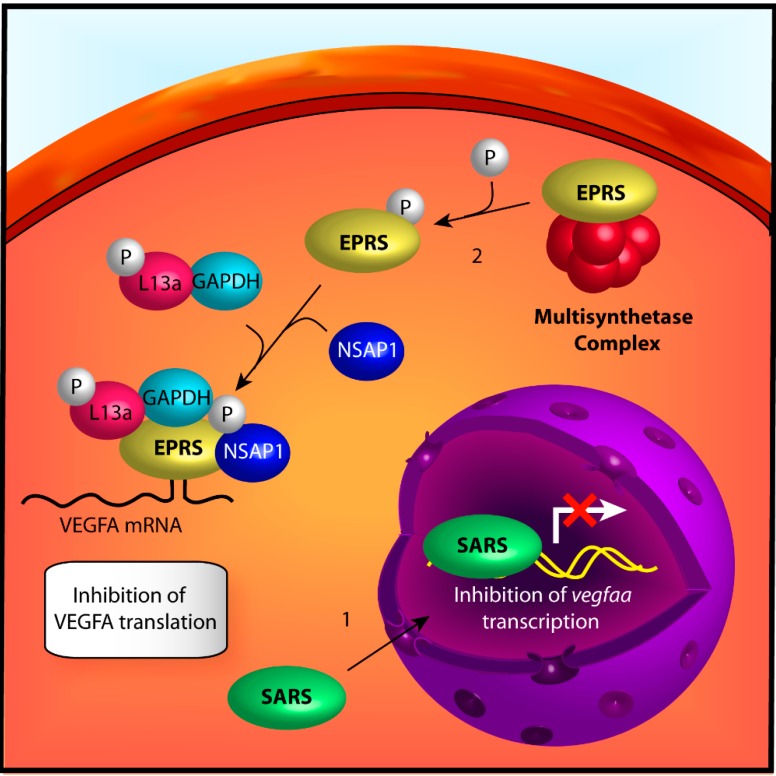
Mechanisms of angiogenesis by intracellularly acting ARS. (**1**) seryl-tRNA synthetase (SARS) is transported into the nucleus via a canonical nuclear localization sequence (NLS) sequence and binds to the vascular endothelial growth factor A gene (*vegfaa*) promoter. The binding of SARS inhibits c-Myc-mediated expression of vascular endothelial growth factor A (VEGFA) through modification of *vegfaa* epigenetics. This effect is angiostatic; (**2**) glutamyl-prolyl-tRNA synthetase (EPRS) is phosphorylated upon treatment of the cell with IFNγ, releasing it from the multisynthetase complex (MSC). Phospho-EPRS binds with L13a, GAPDH, and NSAP1 to form the interferon-γ-activated inhibitor of translation (GAIT) complex. This complex then binds to mRNA containing stem-loop, 3' UTR GAIT elements via EPRS WHEP domains, thereby blocking their translation. As VEGFA contains a GAIT element, this effect is angiostatic.

As part of their role in aminoacylation, ARSs possess features necessary for interactions with nucleic acids. Although normally limited to tRNA, adaptations of this feature can allow for interactions with other molecules bearing the necessary recognition motifs. In prokaryotes, for example, TARS is capable of attenuating translation of TARS mRNA by binding to a tRNA-like secondary structure in the 5' UTR, thereby occluding ribosomal interactions [55]. While this secondary function of TARS has not been observed in eukaryotes, similar mechanisms have been employed by other ARSs in the regulation of angiogenesis. In particular, seryl-tRNA synthetase is able to bind to the *vegfaa* gene promoter and reduce its transcription while glutamyl-prolyl-tRNA synthetase is able to bind to the 3' UTR of VEGFA mRNA and inhibit its translation. In contrast to the intercellular communication propagated by the secreted ARSs, SARS and EPRS mediate intracellular responses to angiogenesis through regulation of canonical angiogenic factors at the transcriptional and translational level (Figure 2).

#### 2.2.1. Seryl-tRNA Synthetase

The understanding of a connection between SARS and angiogenesis appears to have arisen from separate investigations of vascular deficient zebrafish mutants related to the *adrasteia* (*adr*) gene [56] and *ko095* locus [22]. These mutations were associated the dilation of aortic arch vessels (AAV) and the presence of ectopic branching in the cranial and intersegmental vessels (ISV). These effects appeared after 60 and 72 h post fertilization (hpf) for the *ko095* and *adr* mutants respectively. Genetic mapping using select SSLP markers subsequently identified two of the *adr* [57] and one of the *ko095* [22] mutations within the *sars* gene. All three mutations correspond to single nucleotide changes with the *ko095* [22] with one of the *adr* mutants resulting in a premature stop codon and the second *adr* mutant converting a conserved phenylalanine (Phe383) to a valine [57]. In further corroboration of the importance of SARS in vascular development, a third study reported circulation restriction in zebrafish containing *sars* retroviral insertional mutants [58]. The fact that these separately reported mutations in *sars* present with similar deficiencies in vascular development supports the hypothesis that *sars* is the likely source of this phenotype.

Although the above mutations fall within the catalytic core of the enzyme, the effects on the vasculature appear to be unrelated to the canonical aminoacylation function. Knockdown of SARS in endothelial cells using siRNA results in concentration-dependent cell death after 48 h. This effect is attributed to loss of SARS aminoacylation activity and, thus, is non-specific towards cell type. In contrast, SARS knockdown in tube-formation experiments at earlier time points results in increased, albeit disorganized, branching of the tube networks, indicating a endothelial cell-specific function [22]. These results are supported by investigations using the catalytically inactive T429A SARS mutant. Despite its deficiency in aminoacylation injections of T429A SARS mRNA into *ko095* mutant fish completely rescues the aberrant vessel phenotype, thereby separating the two functions [22].

To date, the mechanism through which SARS elicits angiogenesis remains to be elucidated; however, it appears to be dependent on signaling by the VEGFA–VEGFR2 and VEGFC–VEGFR3 pathways. Inhibition of VEGFR2 and 3 with the inhibitors SU5416 or MAZ51, respectively, blocks the dilation of AAVs and ISV ectopic branches. Similar effects are not observed with the EGFR-specific inhibitor AF1478, indicating a VEGF-mediated response [57]. Likewise, knockdown of VEGFA and VEGFC or their respective receptors, VEGFR2 and VEGFR3, in zebrafish by morpholino injection prevents the development of ectopic branches as well [22].

Despite this link to the VEGF receptor family, the necessity of the VEGF ligands for SARS-mediated angiogenesis indicates that the ARS is unlikely to be a direct activator of VEGFR2 or 3. Investigation of mRNA expression changes in vascular-related genes as a result of the *ko095* mutation provides some clarification. Similar to TARS, the *ko095* SARS mutant induces no changes in Notch gene expression while *vegfaa* shows significant increases. Interestingly, the augmented *vegfaa* expression is attenuated by the injection of the aminoacylation-deficient T429A SARS mRNA, suggesting that this effect is due to SARS’s non-canonical function. These findings led to the conclusion that SARS mediates angiogenesis by suppressing VEGFA expression with the dependence on the VEGFC–VEGFR3 pathway explained simply due to the fact that it is essential for angiogenesis in general [22].

Similar to what was observed for YARS, SARSs secondary activity is found to depend on a *C*-terminal, appended domain that arose during vertebrate evolution. This domain, termed UNE-S, encompasses residues 470 to 514 and contains a functional nuclear localization signal (NLS) [23]. Interestingly, both the *ko095* and *adr* non-sense mutations completely abolish the presence of this domain. Similarly, while not located in UNE-S, the vascular-deficient F383V SARS mutant does not localize to the nucleus while the catalytically inactive, but vascular-functional, T429A SARS mutant possesses a clear nuclear distribution. The importance of SARS localization on VEGFA mRNA expression in zebrafish was investigated by knocking down endogenous zebrafish SARS with anti-SARS morpholinos and complementing with mRNA for various SARS constructs. Interestingly, F383V and Δ482–514 SARS (SARS lacking UNE-S domain) exhibit threefold increased expression of *vegfaa* compared to wildtype human SARS and all other constructs with a functional NLS [23]. These results were later recapitulated in both endothelial cells and human embryonic kidney (HEK293) cells in which endogenous SARS was knocked down with shRNA replaced by WT SARS or SARS lacking the NLS sequence (ΔNLS–SARS) [24]. Similarly to zebrafish studies, *vegfaa* expression is increased two-fold in cells treated with SARS shRNA relative to cells treated with control shRNA. Furthermore, SARS knockdown cells complemented with expression of ΔNLS–SARS exhibit a four-fold increase in VEGFA mRNA levels when compared to those complemented with wildtype SARS. As a functional consequence of the increased VEGFA expression, complementation of sh-SARS-treated endothelial cells with ΔNLS–SARS promotes angiogenesis in an endothelial tube-formation assay in contrast to complementation with wildtype SARS. Together, these data suggest a model in which nuclear SARS suppresses expression of *vegfaa* and downstream angiogenesis through a system that is conserved from zebrafish to humans.

Recent investigations have presented a new mechanism for SARS-mediated regulation of *vegfaa* expression [24]. Affinity precipitation of purified SARS with calf-thymus DNA demonstrates that the synthetase is capable of direct interactions with DNA. Chromatin immunoprecipitation (ChIP) and DNA foot-printing analyses narrows this interaction to the *vegfaa* promoter region within −62 to −38 bp from the start site. Of note, this site overlaps with the c-Myc binding site in the *vegfaa* promoter, suggesting that SARS may inhibit the pro-angiogenic activities of c-Myc. Specifically, c-Myc recruits histone acetyltransferase (HAT) proteins to acetylate histones in the *vegfaa* locus, generating transcriptionally active euchromatin [59]. Direct competition between SARS and c-Myc for binding to the *vegfaa* promoter was subsequently demonstrated using an electrophoretic mobility shift assay [24]. Once bound to the DNA, SARS facilitates the formation of heterochromatin through the recruitment of the histone deacetylase SIRT2, thereby counteracting c-Myc-mediated acetylation and inhibiting *vegfaa* transcription.

#### 2.2.2. Glutamyl-Prolyl-tRNA Synthetase

Glutamyl-prolyl-tRNA synthetase (EPRS) is unique among the ARSs due to its bifunctional nature, a characteristic that first originated in higher eukaryotes. The enzyme possesses a modular structure consisting of an *N*-terminal elongation factor 1β-like domain, a glutamyl-tRNA synthetase catalytic domain (EARS), a linker region composed of three tandem WHEP domains, and a *C*-terminal prolyl-tRNA synthetase catalytic domain (PARS) [25]. Additionally, EPRS is associated with the aminoacyl multisynthetase complex (MSC), a heterocomplex composed of nine ARSs (D, EP, I, K, L, M, Q, and R) and three non-catalytic scaffolding proteins (AIMP1-3). Both the WHEP-linker region and association with the MSC contribute to EPRS’s non-canonical functions.

The connection between EPRS and angiogenesis arose from investigations of a phenomenon in which pro-longed exposure of cells to IFNγ induces a marked reduction of translation in specific genes. Termed the interferon-γ-activated inhibitor of translation (GAIT), its mechanism involves the binding of a proteinaceous complex (termed GAIT complex) to stem-loop structures, or GAIT-elements, in the 3'-untranslated region (UTR) of specific mRNA, thereby preventing their translation [26,27]. Yeast three-hybrid and RNA-affinity purification experiments using GAIT element sequences as bait identified the four members comprising the GAIT complex: ribosomal protein L13a (L13a), EPRS, NS1-associated protein-1 (NSAP1), and glyceraldehyde 3-phosphate dehydrogenase (GAPDH) [28,29]. Stimulation of cells by IFNγ results in the phosphorylation and release of EPRS and L13a from the MSC and large ribosomal subunit respectively, and initiates the formation of the active GAIT complex [29]. The binding of the complex to mRNA GAIT elements is primarily mediated by the three tandem WHEP domains in EPRS, emphasizing ARS as a critical component of the GAIT system [25].

The mechanism used by the GAIT complex to inhibit translation was initially determined in studies of ceruloplasmin (Cp) protein expression and then expanded to transcripts associated with other proteins, including VEGF. In Cp, treatment of monocytic cells with INFγ for more than 24 h results in the attenuation of Cp translation. Since the reduction of protein levels are not associated with a concurrent drop in mRNA expression, regulation must occur at the translational level [26]. Binding of the GAIT complex to the GAIT element in the mRNA 3' UTR inhibits the assembly of the 43S ribosome, preventing the translation process [27,28,30]. Using the pattern analysis algorithm software PatSearch several other genes have since been identified with similar predicted 3' UTR GAIT elements, including death associated protein kinase (DAPK), zipper-interacting protein kinase (ZIPK), C–C motif ligand factor-A (CCL22), and VEGFA [31]. In particular, the presence of GAIT elements in VEGFA mRNA suggested the possibility that the GAIT complex could be important for the regulation of angiogenic processes.

To better understand the possible role of the GAIT complex in angiogenesis, the effects of IFNγ-treatment on VEGFA expression and downstream angiogenic activities were investigated with respect to the putative VEGF mRNA GAIT element. The interaction between the GAIT complex and VEGFA mRNA was confirmed using electrophoretic mobility shift assays of ^32^P labeled VEGF riboprobes containing the VEGFA GAIT element. U937 monocyte lysates treated with IFNγ for 16 or 24 h exhibit interactions with all four components of the GAIT complex. Furthermore, the effect of this interaction on VEGF translation was investigated using a luciferase reporter assay. Incubation of luciferase mRNA containing the VEGFA 3' UTR GAIT element with U937 lysate treated with IFNγ for 24 h show a significant reduction in luciferase activity relative to untreated lysate or mRNA lacking the GAIT element. However, translation can be rescued regardless of IFNγ-treatment through the immunodepletion of EPRS, confirming that the suppression was mediated by the GAIT complex. The effect of reduced VEGF levels on angiogenesis was subsequently investigated using endothelial cell proliferation and tube formation assays [32]. Exposure of endothelial cells to conditioned media from U937 monocytes treated with IFNγ for 8 or 12 h exhibits a marked increase in proliferation whereas only basal tube formation is observed with treatments of 16 or 24 h. Similarly, tube formation of endothelial cells cultured on matrigel is enhanced 2.5-fold relative to basal levels in U937 conditioned media treated for 8 h with IFNγ while no change is observed in media treated for 16 or 24 h. These changes in angiogenic activities are consistent with the kinetics of IFNγ-induced activation of the GAIT complex and support a role for the complex in the regulation of angiogenesis.

### 2.3. Regulation of Angiogenesis by ARS-Associated Proteins

Aminoacyl-tRNA Synthetase Interacting Multifunctional Protein 1

**Figure 3:**
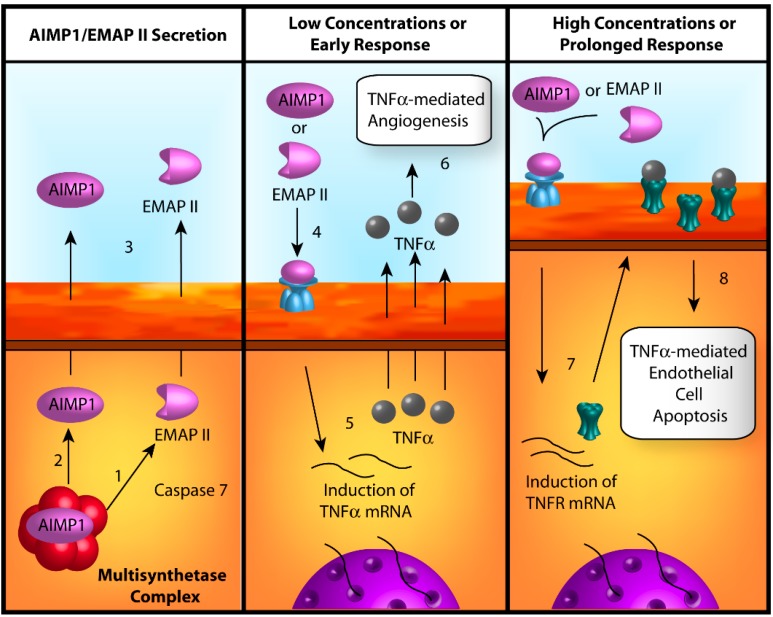
The biphasic responses of aminoacyl-tRNA synthetase interacting multifunctional protein 1 (AIMP1) and endothelial monocyte activating polypeptide II (EMAP II) on angiogenesis. (**1**) Caspase 7 cleaves the *C*-terminal EMAP II peptide from AIMP1, releasing EMAP II from the multi-synthetase complex; (**2**) Alternatively, AIMP1 is released by unknown processes; (**3**) Both EMAP II and AIMP1 are secreted from the cell by mechanisms that remain to be determined; (**4**) At low concentrations (perhaps corresponding to early portions of the AIMP1/EMAP II signaling responses *in vivo*) EMAPII and AIMP1 bind to surface receptors, activating various kinase cascades; (**5**) These kinase cascades induce the expression of tumor necrosis factor α (TNFα); (**6**) TNFα is secreted from the cell and mediates angiogenesis through canonical TNFα pathways; (**7**) At high concentrations (perhaps corresponding to prolonged exposure *in vivo*), AIMP1 and EMAP II induce the expression of the TNFα-receptor (TNFR); and (**8**) Over-stimulation of TNFR by TNFα induces apoptosis through signaling by the TNFR death-domain, resulting in an anti-angiogenic response.

The aminoacyl-tRNA synthetase interacting multifunctional protein 1 (AIMP1; also known as p43) is one of three auxiliary proteins that combines with nine ARSs to make up the MSC heterocomplex. Isolation of the AIMP1 gene from Chinese hamster ovary (CHO) cDNA led to the discovery that the exact sequence for EMAP II, a pro-inflammatory cytokine released from apoptotic cells, is contained within the *C*-terminal region, suggesting that it may serve as the EMAP II precursor molecule and possess potent signaling activity [33]. As indicated above, an EMAP II-like domain is found in a *C*-terminal fragment of YARS and may possess similar signaling functions. During apoptosis, activated caspase 7 during apoptosis catalyzes the cleavage of the *C*-terminal EMAP II domain of AIMP1 and subsequently releases it from the MSC complex (Figure 3) [34]. Once freed, EMAP II is secreted from the cell and stimulates the recruitment of macrophages and initiates other signaling cascades associated with the regulation of inflammation and, of particular interest, angiogenesis.

Both EMAP II and full-length AIMP1 have been observed to regulate angiogenesis in a variety of assays. However, the nature of this regulation is not straightforward as they exhibit both dose-dependent and biphasic characteristics [35]. Low concentrations (0.25 to 1 nM) of either EMAP II or AIMP1 induce endothelial cell migration in transwell migration assays and AIMP1 stimulates angiogenesis in the CAM assay (the effects of EMAP II in CAM assays remain to be investigated). Conversely, concentrations of 10 nM and above exhibit dose-dependent inhibition in the same assay and appear to be related to the induction of endothelial cell-specific apoptosis [35]. Additional studies with EMAP II revealed similar anti-angiogenic responses in both tube formation and rat aortic ring assays at concentrations of 20 µg/mL (0.6 µM) and greater [36]. The cause of this biphasic signaling may be linked to the varying sensitivities of specific kinase cascades [35,37]. Interestingly, full-length AIMP1 appears to be a more potent inducer of migration and apoptosis than the processed EMAP II peptide [35] suggesting a role for the whole protein in signaling events.

Several lines of evidence implicate TNFα in the AIMP1 angiogenic signaling pathway. AIMP1 elicits a striking increase in the expression of TNFα, a potent regulator of both pro- and anti-angiogenic activities [38]. In terms of pro-angiogenic activities, TNFα increases expression of VEGF via NF-κB activation [60] and putatively through the stimulation of YARS and TARS secretion (*vide supra*). Conversely, TNFα can also induce apoptosis in endothelial cells, lending itself to an anti-angiogenic role [61]. AIMP1-stimulated secretion of TNFα initiates the downstream phosphorylation and activation of ERK1/2, a well-known angiogenic signal. The necessity of ERK1/2 activation for AIMP1 regulation of TNFα was subsequently confirmed using the MEK inhibitor U0126, which was shown to attenuate the induction of TNFα [38]. As such, the TNFα activation of ERK1/2 likely contributes to the initial, pro-angiogenic phase of AIMP1 and EMAP II signaling. However, higher concentrations or sustained secretion of AIMP1 or EMAPII enhances the surface representation of TNF receptor 1 (TNFR1) on endothelial cells, thereby facilitating TNFα-induced apoptosis through the TNFR1 death domain [62]. Therefore, TNFα-signaling may contribute to the second, anti-angiogenic portion of AIMP1 and EMAPII’s biphasic response.

In addition to its role in inflammatory signaling, several different studies have revealed that EMAP II is capable of effecting canonical angiogenic pathways. Treatment with EMAP II inhibits endothelial cell migration by disrupting interactions between FN1 and alpha5 beta1 integrins [39]. ELISA-based assays also determined that EMAP II directly interacts with VEGFR1 and 2 and inhibits the binding of VEGF. Functionally, EMAP II inhibition of VEGFRs prevents the phosphorylation of downstream signal mediators, including AKT, ERK1/2, p38MAPK, and Raf as well as VEGF-dependent endothelial cell migration [40]. Furthermore, over expression of EMAP II in cells increases the PSMA7-dependent proteasomal breakdown of HIF1α even under hypoxic conditions and, consequently, reduces hypoxia-induced tube formation in endothelial cells at concentrations of 20 µM [41]. It should be noted, however, that most of the above functions were investigated at EMAP II concentrations that would induce the inhibitory portion of EMAP II’s biphasic response. Therefore, under normal conditions, it is possible that these inhibitory effects would be delayed relative to the initial induction of angiogenesis associated with VEGF- and hypoxia-based signaling.

## 3. Conclusions

### 3.1. Aminoacyl-tRNA Synthetases as Nutrient Sensors

As catalysts for the aminoacylation reaction, ARSs are sensitive to intracellular concentrations of amino acids and ATP, opening the possibility of ARSs to be used as nutrient and energy sensors within the cell. This feature is most apparent in recent studies of leucyl-tRNA synthetase (LARS) in which LARS was found to regulate mTOR-mediated autophagy in a leucine-dependent manner [63,64]. Alternatively, the accumulation of uncharged-tRNA due to amino acid depletion or inhibition of any ARS can activate the GCN2-mediated amino acid starvation response, leading to translational arrest and apoptosis [65]. Since nutritionally starved cells would benefit from the increased vasculature, the acquisition of angiogenesis-related functions by these enzymes would complement their roles as intracellular sensory mechanisms. While no direct nutrient sensing has been observed in angiogenesis-related ARSs, substrate-induced conformational changes are well documented [66,67]. These changes may regulate important interactions responsible for the transport of the ARSs and downstream signaling events essential for their non-canonical activities, and thus warrant further study.

### 3.2. Mechanistic Separation of Angiogenesis-Associated ARSs

Relevant ARSs use a wide variety of mechanisms in their regulation of angiogenesis. However, these enzymes can be subdivided into two broad categories based on the location of their actions. The first, consisting of YARS, WARS, TARS, and AIMP1, function extracellularly and must be secreted from the cell. While little is known of the secretion mechanisms these events are able to accommodate full-length proteins. Since the canonical function of ARSs in translation is catalyzed in the cytoplasm, the export of these proteins likely serves as an important regulatory step in their non-canonical functions. The identification of this system is critical to understanding these secondary functions and their regulatory process.

Conversely, SARS and EPRS regulate angiogenesis intracellularly through interactions with DNA or mRNA [24,25]. Unlike their secreted counterparts, the regulation methods of the intracellular-acting ARSs are better understood. EPRS interacts with mRNA in the cytoplasm and does not require a transport mechanism. Instead, phosphorylation initiates its dissociation with the MSC, thereby acting as a switch between the primary and secondary functions. SARS, on the other hand, interacts with nuclear DNA and must undergo nuclear transport. While the processes regulating this event are not completely known, a nuclear localization signal has been identified within the *C*-terminal domain of SARS that is essential for its regulation of VEGFA transcription. Taken together, these findings would suggest that all of the angiogenic ARSs appear to separate their canonical and non-canonical functions by sequestration. Therefore, undesired angiogenic activity can be limited by regulating the translocation of the ARSs from the cytoplasm or their dissociation from a protein complex.

### 3.3. Associations of Angiogenesis-Related ARSs to Inflammation

Except for SARS, all angiogenesis-associated ARSs possess some connection to inflammation. Both YARS and AIMP1 are associated with the pro-inflammatory EMAP II or ELR domains which activate receptors from the CXC-receptor (CXCR) family. These receptors mediate chemokine signaling pathways important for the coordinated regulation of inflammatory and angiogenic responses during wound healing (reviewed in [68]). Consistent with other ELR containing chemokines, mini-YARS stimulates pro-angiogenic signaling through interactions with CXCR1 expressed by endothelial cells. Interestingly, while all other known ELR chemokines also stimulate CXCR2, knockdown of the receptor has no effect on angiogenesis by mini-YARS, suggesting that mini-YARS may be specific to CXCR1 or possesses yet unknown activities associated with CXCR2. Similarly, EMAPII, as suspected for other ELR-lacking chemokines, regulates angiogenesis through CXCR3. Knockdown of CXCR3 inhibits EMAP II-mediated migration by endothelial cells, a response that would be anticipated for an angiogenic molecule. However, these results appear atypical since CXCR3 is primarily a mediator of angiostasis. Given EMAP II’s biphasic response, it may be possible that both EMAP II’s angiogenic and angiostatic activities are mediated through CXCR3 but are differentially regulated. Additionally, the EMAP II precursor, AIMP1, regulates production of TNFα and its receptor, TNFR, through interactions with CD23. Connections of TNFα to angiogenesis and inflammation are well established in the literature [69]. Of particular interest, TNFα exhibits a dose-response effect, stimulating angiogenesis at lower concentrations and inhibiting it at higher concentrations [70], consistent with the biphasic response observed for EMAP II. Given the sequence homology between EMAP II, similar connections to TNFα may exist with the *C*-terminal domain of YARS and may play an important role in YARS-mediated angiogenesis.

The non-canonical functions of WARS, TARS, and EPRS, appear to be regulated by signals from inflammatory factors rather than initiating them like YARS and AIMP1. Treatment of cells with IFNγ or TNFα stimulates the secretion of WARS and TARS respectively. Additionally, exposure to IFNγ releases EPRS from the MSC, initiating translational control by the GAIT complex. Cross-talk between TNFα and IFNγ are quite common and can be either synergistic or antagonistic [71,72]. In general, IFNγ is anti-angiogenic, consistent with its association with WARS and EPRS signaling. Conversely, the TNFα response to angiogenesis is varied. On one hand, low concentrations of the cytokine appear to mediate the pro-angiogenic effects of EMAP II and stimulate the release of TARS. On the other hand, high concentrations activate death domain-induced endothelial cell apoptosis. Overall, the connection to inflammation underlies a complex association of ARSs with responses to cellular stress separate from their better known, house-keeping roles in translation.

In addition to its secretion by TNFα, TARS is the target of the PL-7 auto-antibody in polymyositis and dermatomyositis autoimmune disorders [73]. While auto-antibodies have been discovered against five other ARSs, TARS remains the only target with documented functions in angiogenesis. Etiological characterizations associated these conditions with auto-reactive T-cells in muscle tissue mediated through two major inflammatory pathways: NF-κB and TNFα signaling [74]. Both of these signaling pathways share connections with inflammation and angiogenesis through regulation of downstream kinases or hypoxia-inducible factor 1α stabilization [75,76]. Of note, muscle biopsies from patients in the early phases of polymyositis and dermatomyositis have demonstrated increased expression of VEGF, possibly due to reduction in capillaries within the affected tissue [77]. However, it is currently unclear as to whether or not the secretion of TARS in angiogenesis has any connection to these diseases or if these autoimmune disorders contribute to neovascularization in tumorigenesis.

### 3.4. The Role of Appended Domains in ARS-Mediated Angiogenesis

Angiogenesis regulatory mechanisms exhibited by the ARSs described in this review are often dependent on appended domains or sequences not found in the prokaryotic enzymes (reviewed in [78]). These features include EMAP II-domains in YARS and AIMP1, an ELR motif in YARS, WHEP domains in WARS and EPRS, and an NLS in SARS. The WHEP domain is exclusive to the ARS family of enzymes and is associated with RNA interactions. The RNA binding affinity of the WHEP domain increases with tandem repeats and is essential for the non-canonical functions associated with EPRS [79]. Conversely, the single WHEP domain of WARS does not have a known RNA-binding function but instead regulates angiogenesis by blocking access of *N*-terminal E-cadherin to the WARS active site. Interestingly, the ELR and EMAP II domains appear to have arisen in insects, whose blood diffuses throughout the body cavity instead of within defined vessels [80]. Likewise, WHEP domains have been observed in organisms as early as choanoflagellates, the closest unicellular ancestor to animals [81]. Therefore, it is likely that these domains developed for a separate purpose and expanded to include angiogenesis following the development of a complex vascular system.

In contrast to the other ARSs, no appended domains have been directly linked to TARS’s secondary functions. Sequence comparisons have identified an *N*-terminal extension (amino acids ~1–80) specific to eukaryotic TARS that is predicted to be unstructured. Its location in the protein places it distal from the only documented, borrelidin-associated conformational change, suggesting that any function this domain may possess is independent of borrelidin’s anti-angiogenic mechanism. Like other unstructured sequences, this domain may serve to facilitate interactions with protein partners essential for the regulation, transport, or other aspects of TARS’s non-canonical function. Nonetheless, the function, if any, of this domain remains to be determined.

### 3.5. ARSs as Targets for Anti-Angiogenic Therapy

Given the importance of angiogenesis in the growth and metastatic progression of several solid tumors, many of the most prominent angiogenic factors, such as VEGF, have been investigated as potential targets for anti-metastatic therapies. Unfortunately, the benefits of these approaches are often temporary owing to adaptive responses by the target tissue [82]. Therefore, better understanding of ARS angiogenic activities may allow for the development of new small molecule inhibitors for use in combinatorial cancer therapies.

The identification of non-canonical activities in ARSs has opened the possibility to utilize these functions for therapeutic benefits. Evidence of this concept can be seen in early investigations of borrelidin’s anti-angiogenic activity where treatment with the compound reduced the occurrence of lung metastases in B16-BL6 mouse models of melanoma at concentrations known to inhibit angiogenesis [83]. While TARS is not specifically mentioned in this study, recent work implicates the synthetase as the sole target of borrelidin, suggesting that the compound’s anti-metastatic properties were likely through its inhibition of TARS [21]. Currently, borrelidin and its derivatives remain the only known anti-angiogenic compounds that target ARSs. However, small molecules targeting other ARS secondary functions have been identified, highlighting the potential utility of ARSs in targeted drug design.

Several natural and synthetic ARS inhibitors have been reported that exhibit anti-microbial or immunosuppressant activity by inhibiting canonical functions of aminoacyl-tRNA synthetases [52,84,85,86]. More relevant to anti-cancer treatments, disruption of an interaction between lysyl-tRNA synthetase (KARS) and the laminin receptor by a small molecule inhibitor was found to inhibit the metastatic progression of a mouse breast cancer model [87]. Considering that chemical intervention of both TARS and KARS non-canonical functions influenced cancer progression it would not be surprising if similar results could be obtained through the manipulation of other angiogenesis-associated ARSs. Therefore, these efforts can be enhanced through the better understanding of regulation and mechanistic processes involved in ARS functions.

### 3.6. Concluding Remarks

The above observations provide clear evidence for a role of ARSs in angiogenesis. Their very different mechanisms suggest that these functions developed separately for each enzyme over the course of evolution. This is not completely surprising as ARSs are among the most ancient and essential enzymes and thus have had a long evolutionary window to acquire secondary functions. Furthermore, their connection to the nutritional and translational status of the cell makes them prime candidates for the development of homeostatic functions, such as angiogenesis, that are essential for the survival of complex, multicellular organisms. Nonetheless, current knowledge still lacks a complete understanding of the molecular mechanisms associated with ARS-mediated angiogenesis. With the exception of EPRS, little is known about the regulatory signals separating the canonical and non-canonical functions. Notably, the lack of a clear secretory mechanism for the extracellularly active ARSs has confounded investigations focused on the initiation of their angiogenesis-associated activities. Furthermore, future drug design would benefit from improvements in knowledge of downstream signaling pathways and structural studies of ARS-partner interactions. Overall, the connection between ARSs and angiogenesis emphasizes the importance of their characterization to the field of vascular biology and opens up new and exciting areas for future research.
